# Intrapulmonary distal airway stem cell transplantation repairs lung injury in chronic obstructive pulmonary disease

**DOI:** 10.1111/cpr.13046

**Published:** 2021-05-07

**Authors:** Xiaofan Wang, Yu Zhao, Dandan Li, Yun Feng, Yusang Xie, Yueqing Zhou, Min Zhou, Yujia Wang, Jieming Qu, Wei Zuo

**Affiliations:** ^1^ East Hospital School of Medicine Tongji University Shanghai China; ^2^ Department of Respiratory and Critical Care Medicine School of Medicine Ruijin Hospital Shanghai Jiao Tong University Shanghai China; ^3^ Kiangnan Stem Cell Institute Zhejiang China; ^4^ Ningxia Medical University Yinchuan China; ^5^ The First Affiliated Hospital Guangzhou Medical University Guangzhou China

**Keywords:** cell transplantation, chronic obstructive pulmonary disease, distal airway stem/progenitor cells, single‐cell sequencing

## Abstract

**Objectives:**

Chronic obstructive pulmonary disease (COPD) is characterized by irreversible lung tissue damage including chronic bronchitis and emphysema, which could further develop into respiratory failure. Many studies have revealed a potential regenerative function of the distal airway stem/progenitor cells (DASCs) after lung injury.

**Materials and Methods:**

Mouse and human DASCs were expanded, analysed, and engrafted into injured mouse lungs. Single‐cell analyses were performed to reveal the differentiation path of the engrafted cells. Finally, human DASCs were transplanted into COPD mice induced by porcine pancreatic elastase (PPE) and lipopolysaccharide (LPS) administration.

**Results:**

We showed that isolated mouse and human DASCs could be indefinitely expanded and were able to further differentiate into mature alveolar structures in vitro. Single‐cell analysis indicated that the engrafted cells expressed typical cellular markers of type I alveolar cells as well as the specific secreted proteins. Interestingly, transplantation of human DASCs derived from COPD patients into the lungs of NOD‐SCID mice with COPD injury repaired the tissue damage and improved the pulmonary function.

**Conclusions:**

The findings demonstrated that functional lung structure could be reconstituted by intrapulmonary transplantation of DASCs, suggesting a potential therapeutic role of DASCs transplantation in treatment for chronic obstructive pulmonary disease.

## Graphical abstract

Mouse and human DASCs could be indefinitely expanded and were able to further differentiated into mature alveolar structures during in vitro cultures. Single‐cell analysis indicated that the engrafted cells expressed typical cellular markers of type I alveolar cells as well as the specific secreted proteins. Transplantation of human DASCs derived from chronic obstructive pulmonary disease (COPD) patients into the lungs of NOD‐SCID mice with COPD repaired the tissue damage and improved the pulmonary function.

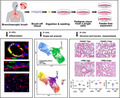

## 1 INTRODUCTION

Chronic obstructive pulmonary disease (COPD) is a disorder that comprises features of chronic bronchitis and/or emphysema, which can eventually lead to cor pulmonale and respiratory failure.^1^ The high morbidity and mortality have made COPD the third leading threat of global public health, affecting more than 700 million people worldwide.^2^, ^3^, ^4^, ^5^ Patients with COPD experience persistent airflow limitation, decreased gas diffusing capacity, disabling symptoms, and exacerbations caused by structural destruction of the lung parenchyma.

Due to the lack of known etiology and full understanding of its pathogenesis, there has been no effective treatment for COPD so far.^6^ Current therapeutic approaches improve the airflow limitation but show little benefit in preventing the alveolar structural destruction or air‐exchanging capacity decline. Therefore, it is urgent to develop a new treatment for COPD.^7^, ^8^ In recent years, a growing body of evidence has shown that tissue regeneration could be applied in the treatment of various lung diseases.^9^, ^10^, ^11^ In a general way, the proliferation of differentiated cells and/or the deployment of specialized stem cells/progenitor cells could achieve tissue regeneration, which has been used in some previous studies.^12^, ^13^ Adult lung tissues experience a potent regenerative process after injury, which is dominated by the activation of a rare stem/progenitor cell population present in the basal layer of the distal airway.^14^, ^15^ This cell population is identified as distal airway stem cells (DASCs) expressing transcription factor p63 and keratin‐5 (Krt5).^16^, ^17^, ^18^, ^19^ After lung injury, sporadic DASCs can rapidly re‐enter into the cell cycle, followed by migration and differentiation into mature bronchial and alveolar epithelium, which eventually leads to lung tissue regeneration.^17^ The feasibility of large‐scale in vitro expansion and competent regenerative capacity have made DASC an ideal candidate for cell therapy.^18^, ^19^, ^20^ Although the onset and progression of DASC‐dominant lung regeneration have been studied in some acute lung injury animal models, such as mice models induced by the H1N1 influenza infection or bleomycin treatment, how DASCs respond to the lung damage under chronic pathological condition is largely unknown. In this study, we aimed to determine whether DASC transplantation has a beneficial effect on COPD induced by porcine pancreatic elastase (PPE) and lipopolysaccharide (LPS) administration. The results showed that DASCs could alleviate pulmonary inflammation and emphysema in the lung. In addition, the transplantation improved the air exchange function of mice with COPD, compared with control groups. In conclusion, DASCs transplantation might serve as a promising therapeutic approach for COPD patient treatment.

## 2 MATERIALS AND METHODS

### 2.1 Animals

Male and female NOD‐SCID mice (6‐8 weeks), weighing 16‐18 g, and C57/B6 mice (6‐8 weeks), weighing 16‐22 g, were used. All mice were maintained in specific‐pathogen‐free conditions in the Center of Laboratory Animal, Tongji University until euthanization. All animal‐related procedures were conducted in strict compliance with approved protocols and under the guidance of the Animal Protection and use Committee of Tongji University.

### 2.2 Isolation and culture of DASCs from mouse and human lung tissues

The lung tissues of adult mice were soaked in ice‐cold washing buffer (F12 medium with 1% Pen/Strep, and 5% FBS). The trachea and two main bronchi were separated from the lungs, and the lobes of the lungs were cut into small pieces and gently shaken overnight. Then the tissues were digested with a dissociation buffer (F12/DMEM medium with 1 mg/mL protease, 0.01% trypsin, and 10 ng/mL DNase I). The isolated cells were washed with cold F12 medium and passed through 70 μm Nylon mesh, and then inoculated into irradiated 3T3 feeder layer cells. Under the culture condition of 7.5% CO_2_, the mouse DASC colonies appeared after 3‐5 days of culture.

For human DASC culture, patients with chronic lung diseases (COPD) were diagnosed by ATS/ERS criteria. All individuals went through a thorough medical examination before sampling. The bronchoscopic procedure for sampling was performed by respiratory physicians using a flexible fibre‐optic bronchoscope (Olympus, Japan). Before the bronchoscopy, oropharyngeal and laryngeal anaesthesia was obtained. After the bronchoscope was advanced through the vocal cords, 2 mL of 2% lidocaine solution was instilled into the trachea and both main bronchi through the working channel of the bronchoscope. Then a disposable 2 mm brush was advanced through the working channel of the fibre‐optic bronchoscope and used to collect airway epithelial cells by gently gliding the brush back and forth 1 or 2 times in random regions of the 4~5th order bronchi. All the human tissues were obtained following clinical SOP under the patient’s consent and approved by Shanghai East Hospital Ethics Committee (Shanghai, China).

To isolate the human DASCs, brush with samples was cut into 1 cm pieces. After removing sputum, the brush pieces were directly digested with dissociation buffer. Specimens were incubated at 37°C for an hour with gentle rocking. Dissociated cells were passed through 70 μm Nylon mesh and then washed twice with cold wash buffer. Cell pellets were collected and plated onto mitomycin‐inactivated 3T3 feeder cells in a culture medium as previously described.^18^

To obtain a single cell‐derived clone, cells are digested into single cells, loaded through 40 μm Nylon mesh and seeded with extremely low density, then a single colony grown up from a single cell was picked up by clone cylinder (Sigma, USA) and high vacuum grease after its neighbouring colonies were cleared by scraper to ensure the pedigree purity.

For labelling of cells by GFP, pLenti‐CMV‐EGFP plasmid was transfected into 293T cells together with lentiviral packaging mix (Life Technologies, USA). Lentivirus supernatant produced by 293T was collected, filtered, and cryopreserved before use. To infect DASCs, 0.5 mL lentivirus containing medium was directly added to 2 mL cell culture medium with 10 μg/mL polybrene and incubated for 12 hours.

### 2.3 Differentiation of DASCs in vitro

#### 2.3.1 3D Matrigel culrue

Mouse DASCs were implanted on Matrigel Matrix (Corning, USA) as described earlier.^16^ The cells were cultured in a serum‐free medium for seven days, and fibroblast growth factor 10 (50 ng/mL, PeproTech), transferrin (5 μg/mL, PeproTech), hepatocyte growth factor (20 ng/mL, PeproTech), 2% Matrigel, and 5% bovine serum albumin were added. The differentiated culture was embedded in O.C.T. In the complex, the differentiated cells were identified by immunofluorescence staining.

#### 2.3.2 Air‐liquid interface (ALI) culture

DASCs were differentiated on ALI cultures. Briefly, cells were cultured on Transwell plates (Corning, USA). At confluence, the medium in the upper chamber was removed and new medium was added to the lower chamber to create an air‐liquid interface. Retinoid acid (50 nM), BSA (1 mg/ml) and transferrin (5 mg/mL) were included in culture medium. After 16 days of differentiation, the epithelial structures along with Transwell membrane were collected for paraffin section and immunostaining.

#### 2.3.3 Monolayer differentiation culture

Cells were plated on 12‐well plates (10^4^ cells per well) pre‐coated with 20% Matrigel (Corning, USA). 24 hours later the culture medium was changed to a serum‐free differentiation medium for five days. Then cells were harvested, fixed and immunostained with HOPX antibody or IgG control. An image‐based cell counter (Countess II FL, Thermo Fisher Scientific, USA) with a fluorescent filter set were used to determine the HOPX+ cell frequency.

### 2.4 Differentiation of DASCs in vivo

6‐8 weeks C57/B6 mice were used for in vivo DASCs differentiation (30‐dpt group: n = 3, 90‐dpt group: n = 4). Bleomycin was intratracheally administrated to isoflurane‐anaesthetized mice at a concentration of 3U/kg seven days prior to transplantation. DASCs suspended in PBS (10^6^ cells per mouse) were transplanted via the trachea. At different time points, mice were sacrificed and their lung tissues were harvested to detect GFP signal by fluorescence stereomicroscope (MVX10, Olympus, Japan), the region of which was dissected for further work.

Single cell suspensions were generated as follows. Firstly, the GFP+ tissues of the lung were collected and immersed in a cold F12 medium with 5% FBS, followed by being minced into small pieces and digested with dissociation buffer on a shaker for 1~1.5 hours. Dissociated cells were filtered through 100 μm cell strainers, and Red Blood Cell Lysis Buffer was used to remove erythrocyte. Cell pellets were resuspended in DMEM containing 1% FBS following washing twice, and then passed through 30 μm strainers. Sorting and subsequent quantification were performed on BD FACS Aria ll cytometers.

### 2.5 ScRNA‐seq data analysis from DASC differentiated in vivo

Single cells were captured and barcoded in 10× Chromium Controller (10× Genomics). Subsequently, RNA from the barcoded cells was reverse‐transcribed and sequencing libraries were prepared using Chromium Single Cell 3’v3 Reagent Kit (10× Genomics) according to the manufacturer’s instructions. Sequencing libraries were loaded on an Illumina NovaSeq with 2 × 150 paired‐end kits at Novogene, China. Raw sequencing reads were processed using the Cell Ranger v.3.1.0 pipeline from 10× Genomics. In brief, reads were demultiplexed, aligned to the human GRCh38 genome and UMI counts were quantified per gene per cell to generate a gene‐barcode matrix. Data were aggregated and normalized to the same sequencing depth, resulting in a combined gene‐barcode matrix of all samples.

The following criteria were then applied: gene number between 200 and 7000 and mitochondrial gene percentage <0.3. Genes were filtered out that were detected in less than three cells. Finally, a filtered gene‐barcode matrix of 30‐dpt and 90‐dpt sample was integrated with Seurat version 3 to remove batch effects across the different samples. In parameter settings, the first 30 dimensions of canonical correlation analysis (CCA) and principal‐component analysis (PCA) were used.

The filtered gene‐barcode matrix was first normalized using the ‘LogNormalize’ method in Seurat version 3 with default parameters. The top 2000 variable genes were then identified using the ‘vst’ method in Seurat FindVariableFeatures function. A linear transformation (ScaleData) was applied as a pre‐processing step and PCA was performed using the top 2000 variable genes. We then selected the top 30 significant PCs for two‐dimensional uniform manifold approximation and projection (UMAP). We used FindCluster in Seurat to identify cell clusters. Immune cell clusters were removed and the other cells were re‐clustered using the same parameter mentioned above in the clustering step and parameter resolution was set to 0.1. To identify the marker genes, differential expression analysis was performed by the function FindAllMarkers in Seurat with the likelihood‐ratio test. Differentially expressed genes that were expressed at least in 25% cells within the cluster and with a fold change of more than 0.25 (log scale) were considered to be marker genes. Featureplots were performed by the function Featureplot in Seurat with the default parameters.

To infer the cluster and lineage relationships between the cell types identified, Monocle3 was used. UMAP embeddings and cell clusters generated from Seurat were used as input, and trajectory graph learning and pseudotime measurement through reversed graph embedding were performed with Monocle3.

Gene Ontology (GO) enrichment analysis of differentially expressed genes was implemented by the ClusterProfiler R package. Dot plots were used to visualize enriched terms by the ggplot2 R package.

### 2.6 Transplantation of DASCs into COPD mouse

6‐8‐week‐old NOD‐SCID mice were purchased from Shanghai SLAC Experimental Animal Co., Ltd. Mice were anaesthetized with isoflurane, the lung was injured by intratracheally instilled with LPS (20 μg/mL) and PPE (8 U/mL) in the volume of 50 μL on day 0, 1, 2 for 3 consecutive days and the weight of the mice was recorded every day afterward. 2 × 10^6^ human DASCs or PBS in a volume of 40 μL were transplanted into the mouse lung on day 5. Intratracheal delivery was performed by injecting the cells into the trachea via the mouth which was described in our previous publications.^16^, ^17^, ^18^, ^19^ The DASCs were counted, washed and resuspended by PBS before transplantation.

### 2.7 The wet/dry lung weight ratio

To quantify the magnitude of pulmonary emphysema caused by PPE/LPS administration, we evaluated the wet/dry (W/D) ratios. The lungs were excised on indicated days after the injury, and the wet weight was recorded. The dry weights were obtained after the lungs were dried in an oven at 70°C for 72 hours. The W/D ratios were then calculated.

### 2.8 Tissue histology

After euthanization, the thoracic cavity was carefully cut open without touching the lung. In situ fixation was performed by injecting 3.7% formaldehyde through the trachea using a 29 G needle. Direct fluorescent image of the chimeric lung was acquired under a fluorescence stereomicroscope (MVX10, Olympus, Japan). For paraffin sectioning, the lung was dissected and further fixed in 3.7% formaldehyde at 4°C overnight. The tissue was dehydrated by gradient ethanol and processed in an automatic tissue processor, then embedded into the paraffin blocks. All the samples were sliced into 5 μm thickness using a microtome (Leica microsystem, Germany). Haematoxylin eosin staining was carried out according to the standard procedure. The pathological changes of airway inflammation and pulmonary vascular remodeling were observed after H&E staining. Lung injury scores are calculated as described earlier.^21^

### 2.9 Immunofluorescence

Immunofluorescence staining was conducted by standard protocol. Antibodies used in the current study were: anti‐Krt5 (1:200, EP1601Y, Thermo Fisher Scientific, USA), anti‐p63 (1:500, 4A4, Abcam, USA), Ki67 (1:500, B126.1, Abcam, USA), AQP5 (1:500, EPR3747, Abcam, USA), HOPX (1:200, ab230544, Abcam, USA), PDPN (1:200, 18H5, Santa Cruz Biotechnology, USA), CC10 (1:200, T‐18, Santa Cruz Biotechnology, USA) and LAMP3 (1:200, 12632‐1‐AP, Proteintech, USA). Alexa Fluor‐conjugated 488/594 (1:500, Life Technologies, USA) antibodies were used as secondary antibodies. The stained slides were stored in 4°C darkness and photographed with a fluorescence microscope (Nikon80i and Eclipse Ti, Nikon, Japan).

### 2.10 Arterial blood gas measurement

Mice were anaesthetized and the blood samples were drawn from the carotid aorta into polypropylene syringes containing 60 IU of dry, electrolyte‐balanced heparin (PICO70; Radiometer Medical, Copenhagen, Denmark). Partial oxygen pressure (pO2), partial carbon dioxide pressure (pCO2) and oxygen saturation (sO2) were measured by using ABL90 Flex Blood Gas Analyzer (Radiometer Medical).

### 2.11 Statistics

All statistical analyses were performed using GraphPad Prism version 7 software. All experiments were performed in three independent triplicates at least.

## 3 RESULTS

### 3.1 Isolation and identification of mouse and human DASCs

**FIGURE 1:**
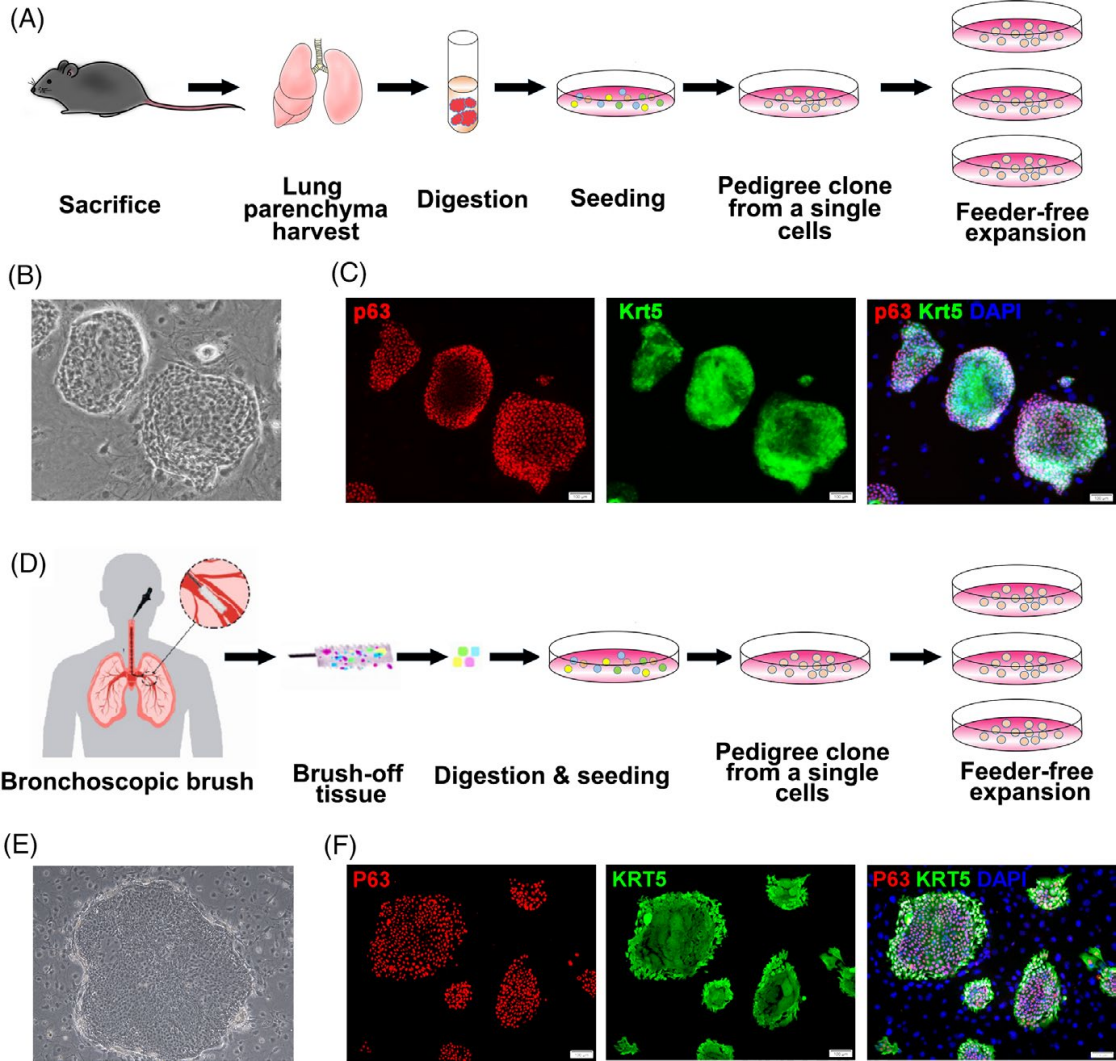
Isolation, expansion and identification of mouse and human DASCs. (A) Schematic illustrating the process of mouse DASCs in vitro culture. (B) Representative bright‐field image of clonogenic mouse DASCs on feeder cells. (C) Clonogenic mouse DASCs immunostained with Krt5 and p63. Scale bars, 100 μm. (D) Schematic illustrating the process of human DASCs culture. (E) Representative bright‐field image of clonogenic human DASCs on feeder cells. (F) Clonogenic human DASCs immunostained with KRT5 and P63. Scale bars, 100 μm

Mouse DASCs were isolated from mouse lung parenchyma (Figure 1A). The clonality of these cells was validated by a single‐cell derived cloning assay, which showed the stable passage with a typical epithelial progenitor/stem cell morphology (Figure 1B). The stemness of mouse DASCs was confirmed by expression of key markers Krt5 and p63 (Figure 1C). Similarly, human DASCs derived from bronchoscopic brushed‐off tissue from the 4~5th order bronchi of COPD patients were successfully cloned and further propagated (Figure 1D and Table S1). The morphology and key marker expression patterns of human DASCs were similar to their counterpart in mice (Figure 1E and 1F). No significant differences were observed among cells from different individuals regarding the clone number, cell morphology, and marker expression levels.

**FIGURE 2:**
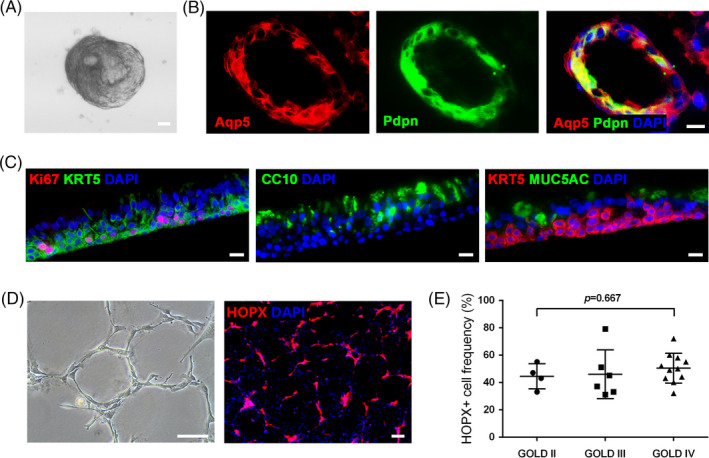
Differentiation of mouse and human DASCs in vitro. (A) Representative bright‐field imaging of mouse DASCs in 3D Matrigel culture. Scale bar, 20 μm. (B) Immunofluorescence of 3D Matrigel culture of mouse DASCs with the expression of AT1 cell markers Aqp5 and Pdpn on Day 10. Scale bar, 20 μm. (C) Immunostaining on paraffin‐embedded sections of ALI differentiation cultures of human DASCs with indicated markers on Day 16. Scale bars, 20 μm. (D) Representative bright field and immunostained images of human DASCs in the monolayer differentiation culture on Day 7. HOPX is an AT1 cell marker. Scale bars, 100 μm. (E) Quantification of the HOPX+ cell frequency of differentiated DASCs isolated from COPD patients with different COPD severity (GOLD) status. Horizontal lines represent the mean within groups, with standard deviation error bars

In order to comprehensively evaluate the differentiation capacity of the cultured DASCs, cells were subjected to multiple differentiation assays. In a 3D matrigel culture system, DASCs spontaneously aggregated and formed an alveolar‐like spherical structure, which was composed of differentiated cells expressing type I alveolar cell markers Aqp5 and Pdpn (Figure 2A and 2B). Further, we tested if DASCs could regenerate bronchial epithelium in an air‐liquid interface (ALI) system. As shown in Figure 2c, the differentiated cells formed a stratified epithelium structure marked by secretory cells (CC10+/MUC5AC+) and proliferating basal cells (KRT5+/Ki67+). The differentiation capacity of human DASCs was also examined in a monolayer differentiation system. Cultured human DASCs gradually formed the alveoli‐like structures lined by elongated cells expressing mature type 1 alveolar cell marker HOPX (Figure 2D). It is worth mentioning that the HOPX+ cell ratio was assessed for each COPD patient. No significant difference was observed in HOPX+ cell frequency among patients of different disease severity (classified according to the GOLD standard) (*P* = 0.667, Figure 2E). Taken together, our data showed that DASCs from COPD lungs still maintained the robust clonality and differentiation capacity.

### 3.2 Transplanted DASCs gave rise to alveolar type I cells and proliferating DASCs in vivo

**FIGURE 3:**
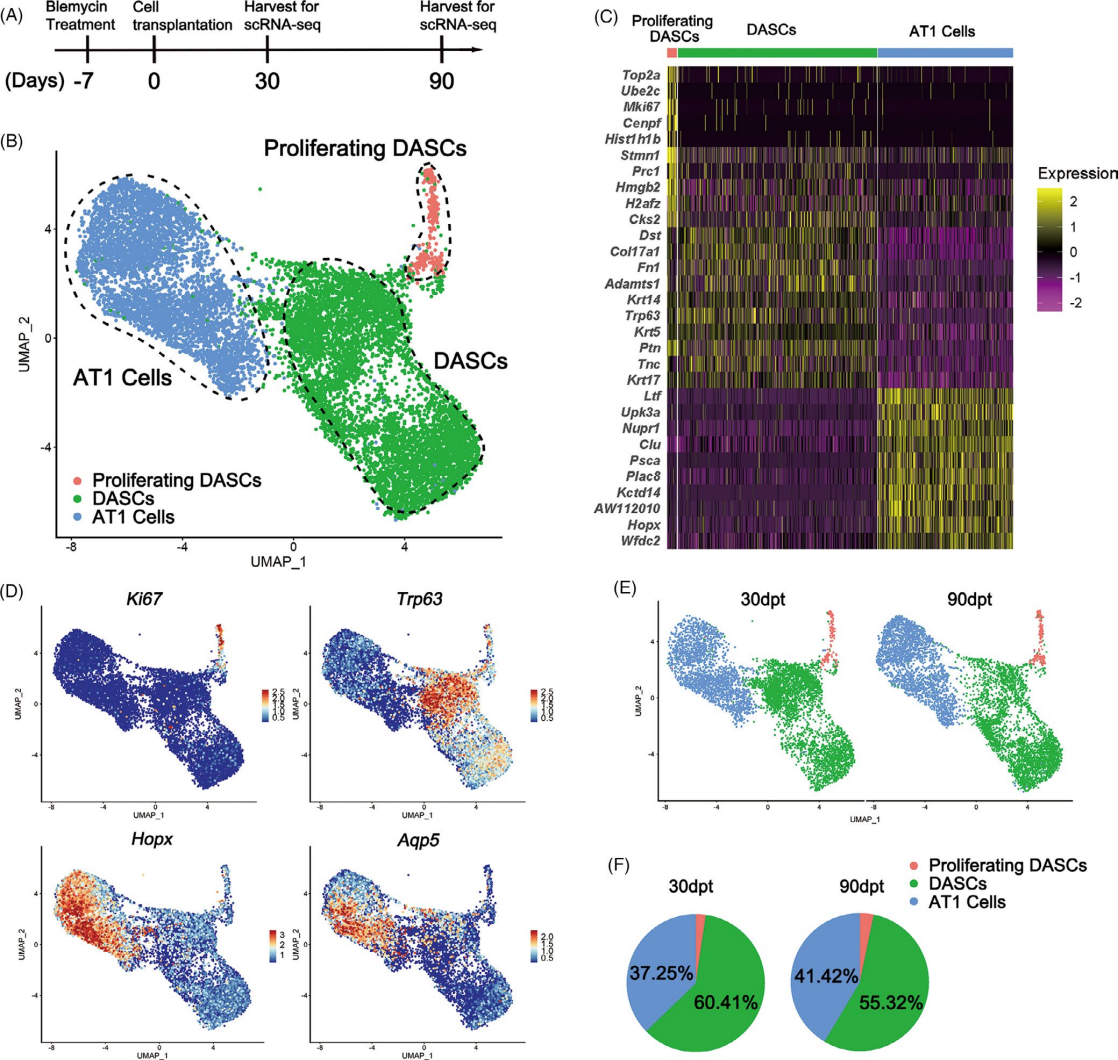
Single‐cell RNA sequencing revealed the differentiation of mouse DASCs in vivo. (A) Schematic showing procedure of single‐cell RNA sequencing analysis. (B) UMAP plot of all engrafted cell clusters 30‐dpt and 90‐dpt. dpt, day post‐transplantation. (C) Heat map of the top ranked genes highly expressed in each cluster. Colour scheme is based on z‐score distribution from –2 (purple) to 2 (yellow). (D) Feature plots showing distinct populations of cells with specific marker expression. Trp63 = p63. (E) UMAP plots of engrafted cell among 30‐dpt and 90‐dpt sample, respectively. (F) Pie plots showing the percentages of distinct cell clusters in 30‐dpt and 90‐dpt sample

To further investigate DASCs differentiation in vivo, we intratracheally transplanted GFP‐labelled mouse DASCs into the lung of mice that received bleomycin injection (Figure 3A). After 30 and 90‐day‐post‐transplantation (dpt), the lungs were harvested and digested into single cells. Then the engrafted GFP+ cells were sorted and subjected to the following scRNA‐seq analysis. In total, we sorted and sequenced 15 447 cells (30‐dpt = 8471, 90‐dpt = 6976) from the transplanted lungs. By using the same stringent quality controls, 11 629 cells (30‐dpt = 5766, 90‐dpt = 5863) were eventually analysed (Figure S1). Clustering analysis of the sequencing data identified three distinct cell populations (Figure 3B), with their unique gene expression cluster (Figure 3C). According to the expression levels of Krt5 and Ki67, the three cell clusters were then annotated as undifferentiated Krt5+/p63+ DASCs, Krt5+/Ki67+ proliferating DASCs, and Hopx+/Aqp5+ type 1 alveolar (AT1) cells, respectively (Figure 3D). The key marker expression was further confirmed by immunostaining (Figure S2). Interestingly, the 90‐dpt engrafted cells showed a larger AT1 cell population, and a smaller DASC population compared to the 30‐dpt engrafted cells, while the proportion of proliferating DASCs remained unchanged (Figure 3E and 3F).

**FIGURE 4:**
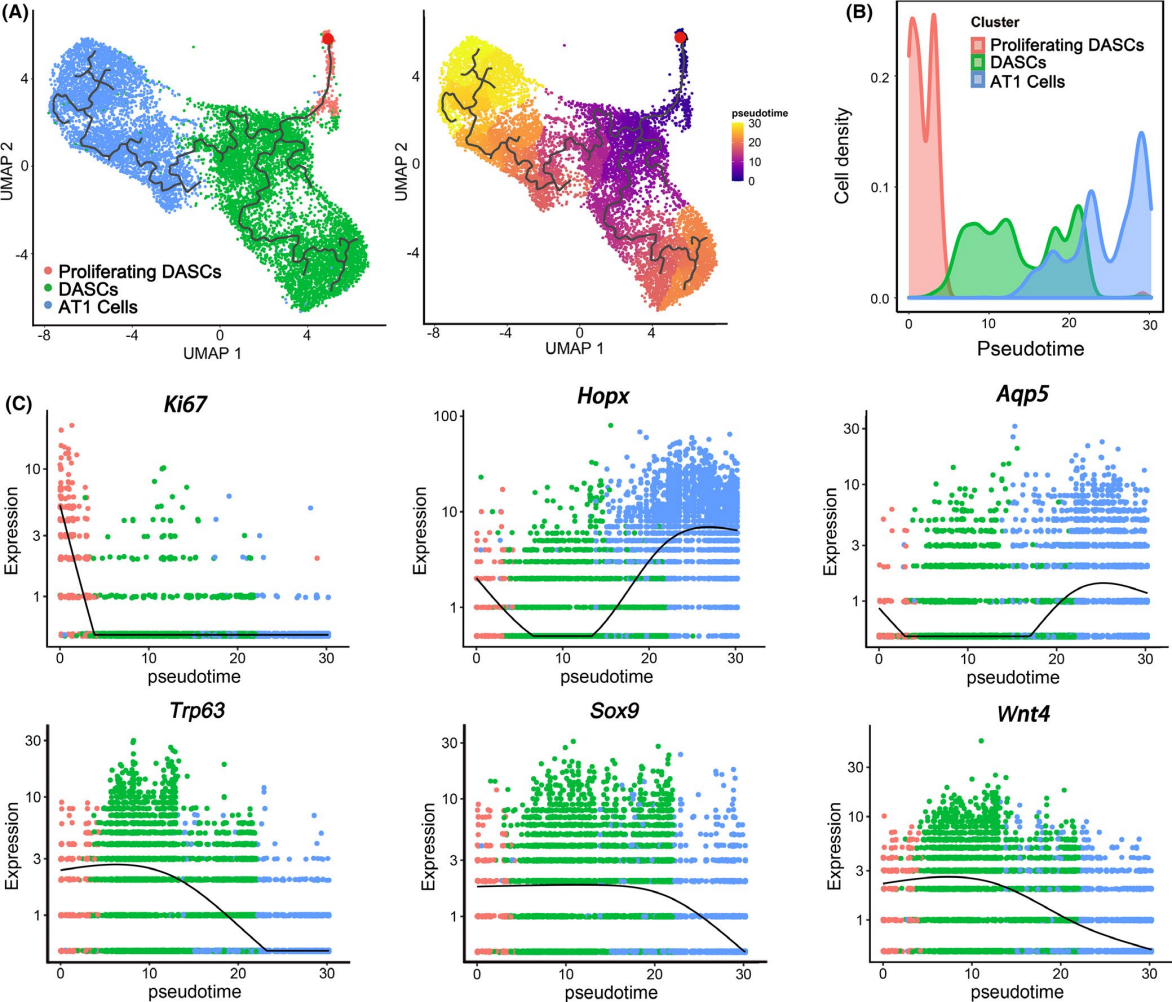
Transplanted DASCs give rise to AT1 cells and proliferating DASCs in vivo. (A) Pseudo‐time trajectory projected onto a UMAP of all engrafted cells. The red dot indicates the start point of the trajectory. Pseudo‐time values are colour coded. (B) Cell density of three clusters along inferred cellular trajectory reflecting differentiation process. Cells are ordered by pseudo‐time (cell‐to‐cell distance metric). (C) Expression of specific genes along the cell trajectory important for the differentiation paths. The dots indicate the gene expression of individual cells. Trp63 = p63

Next, monocle pseudotime analysis indicated that the transplanted DASCs could give rise to Krt5+/Ki67+ proliferating DASCs and Hopx+/Aqp5+ AT1 cells, respectively, which was in line with the in vitro differentiation data (Figure 4A and 4B). Expression pattern of Ki67, Hopx, and Aqp5 indicated two different directions along the cell trajectory (Figure 4C). Furthermore, we found that Notch signalling pathway (p63) and Wnt signalling pathway (Sox9/Wnt4) were involved in the differentiation of transplanted DASCs, consistent with previous findings that Notch and Wnt signalling were critical for lung regeneration (Figure 4C). Altogether, the data revealed that after intratracheal transplantation, the exogenous DASCs incorporated in the injured lung and differentiated into alveolar type I cells and proliferating DASCs.

**FIGURE 5:**
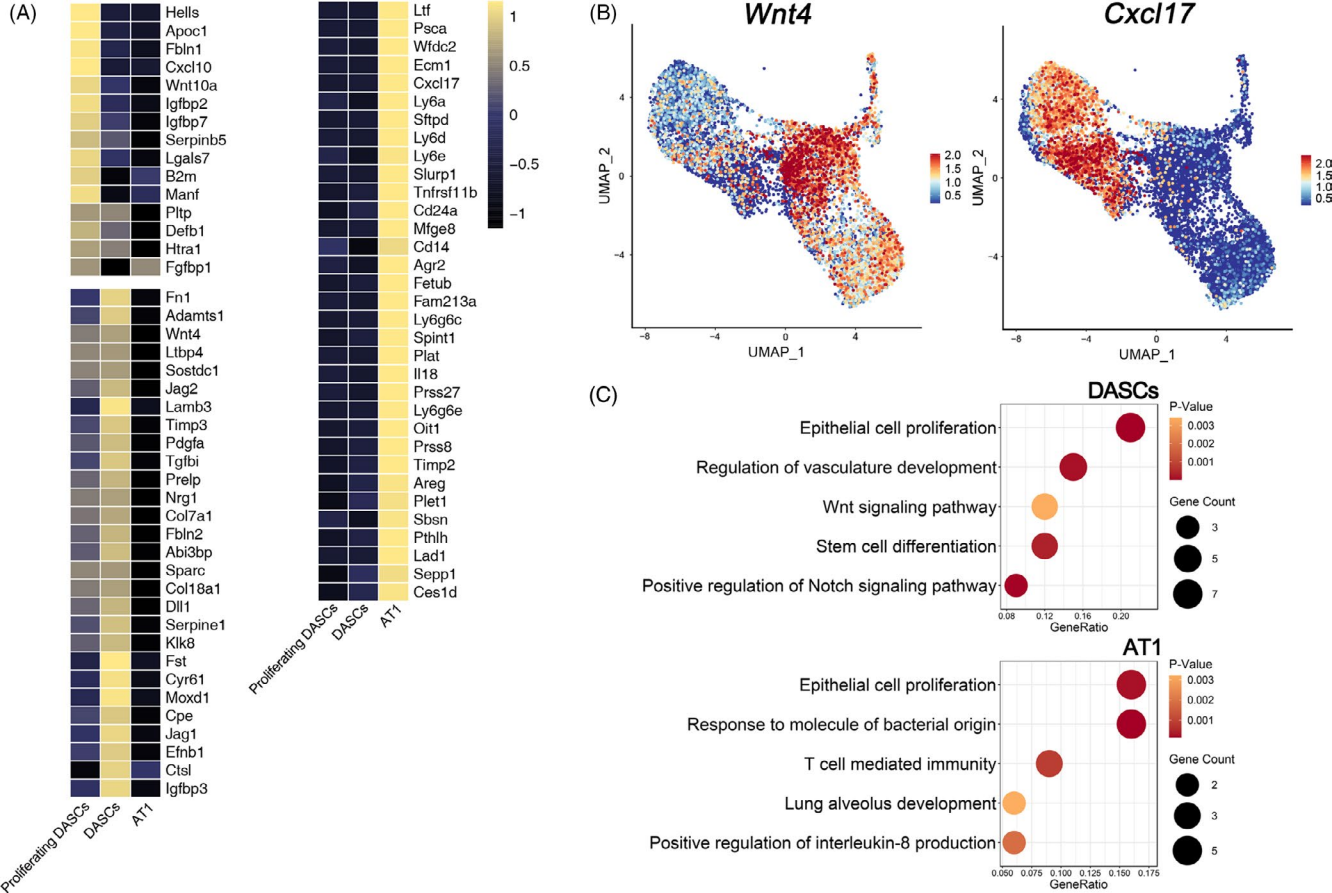
DASC Secretome Gene Analysis. (A) Heatmaps showing single cell–type specific average expression of secretome gene. Mean expression values of the genes were calculated in each cluster. In the heatmap, each row represents one gene, and each column is a single cell type. (B) Visualization of cell‐type‐specific secretome gene expression. (C) Gene Ontology enrichment analysis of the differentially expressed secretome genes expressed in three cell types

We next analysed the secretome of three cell types generated after DASC transplantation in vivo. 1272 genes were analysed that are predicted to encode secreted proteins according to a previously published single‐cell dataset.^22^ Among them, 790 genes were detected by mRNA levels and 76 genes were specifically expressed in the three cell types. The following clustering analysis revealed that each cell type exhibited a specific secretome pattern (Figure 5A). For example, Wnt4, which is important for epithelial cell proliferation and lung development,^23^ was enriched in the DASC cell cluster, whereas Cxcl17, which exhibits anti‐microbial and anti‐inflammation functions,^24^ was abundantly expressed in the AT1 cell cluster (Figure 5B). Furthermore, ontology enrichment analysis revealed that genes relevant to the processes of epithelial cell proliferation, vasculature development and stem cell differentiation were enriched in DASCs. However, genes engaged in lung alveolus development, response to molecule of bacterial origin, and positive regulation of interleukin‐8 production were enriched in AT1 cells, indicating their roles in resisting bacteria and eliminating inflammation (Figure 5C).

### 3.3 Establishment of a mouse disease model for COPD

**FIGURE 6:**
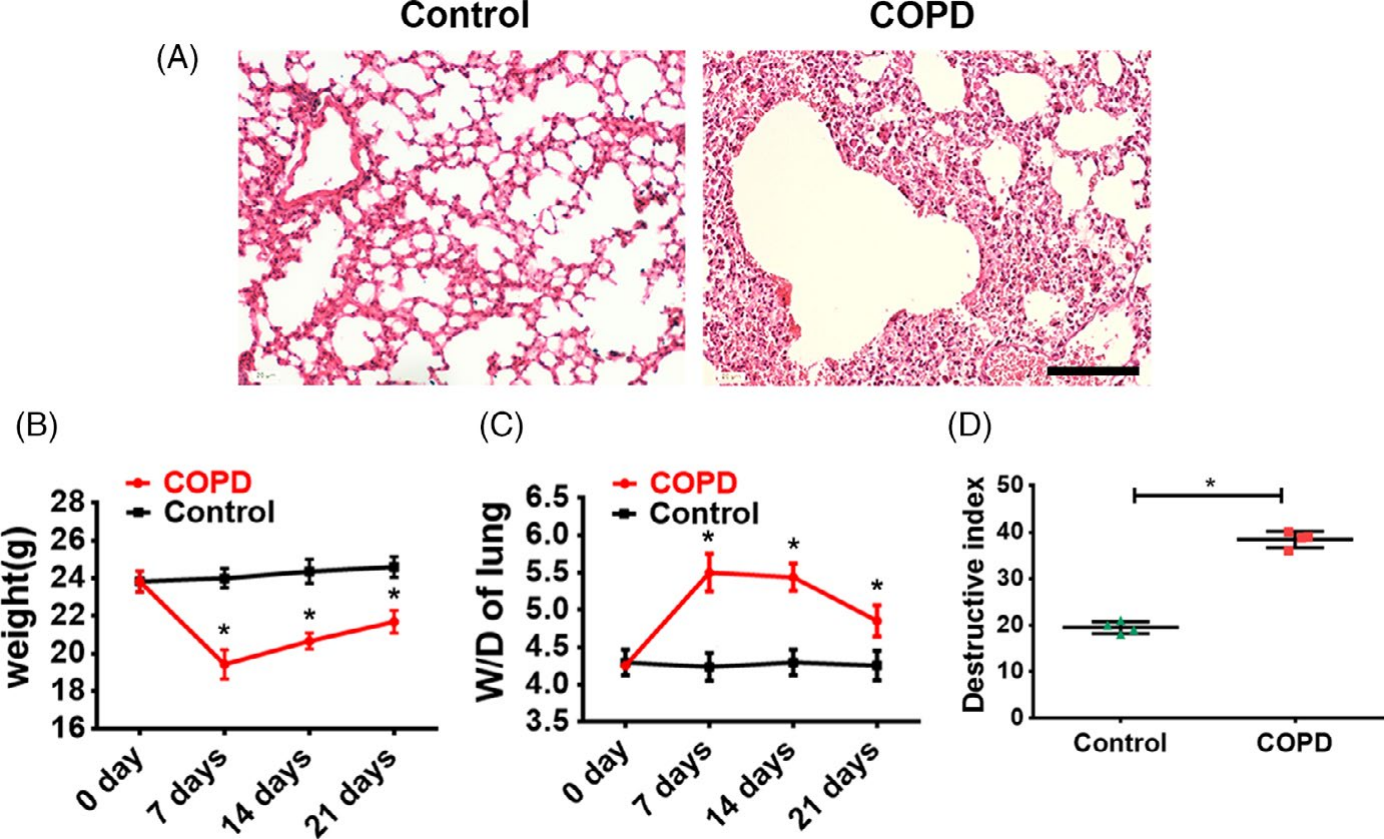
COPD mice model induced by PPE and LPS administration. (A) Representative H&E staining of the lung harvested on Day 7 after PPE/LPS treatment. Scale bar, 100 μm. (B) The weight changes of COPD mice and untreated control mice. N = 4. Error bars, SEM *P < .05. (C) The changes of wet/dry (W/D) lung weight ratio of COPD mice and untreated control mice. N = 4. Error bars, SEM *P < .05. (D) The lung destructive index of COPD mice and untreated control mice on Day 7. n = 4. Error bars, SEM *P < .05

A COPD mouse model was set up by intratracheal instillation of PPE to induce emphysema, combined with LPS administration to induce pulmonary inflammation, which altogether mimicked the COPD pathological process in human patients. Histology analysis showed that seven days after the PPE/LPS administration, obvious immune cell infiltration, vascular congestion and alveolar collapse were observed in the COPD mice, while the lungs in the untreated control mice showed a normal structure (Figure 6A). The body weight significantly decreased (*P* < .05, Figure 6B) while the wet/dry lung weight ratio (W/D) was significantly elevated (*P* < .05, Figure 6C) in COPD mice at 7, 14 and 21 days after PPE/LPS delivery, compared with the untreated controls. Moreover, COPD mice showed a more severe lung structure damage, as assayed by the destructive index of the lung measured by the percentage of the damaged area (Figure 6D). All the above results have indicated that the COPD mice experienced serious pneumonedema and severe lung parenchymal injury.

### 3.4 Pulmonary structure and function rescued by DASC transplantation in COPD mice

**FIGURE 7:**
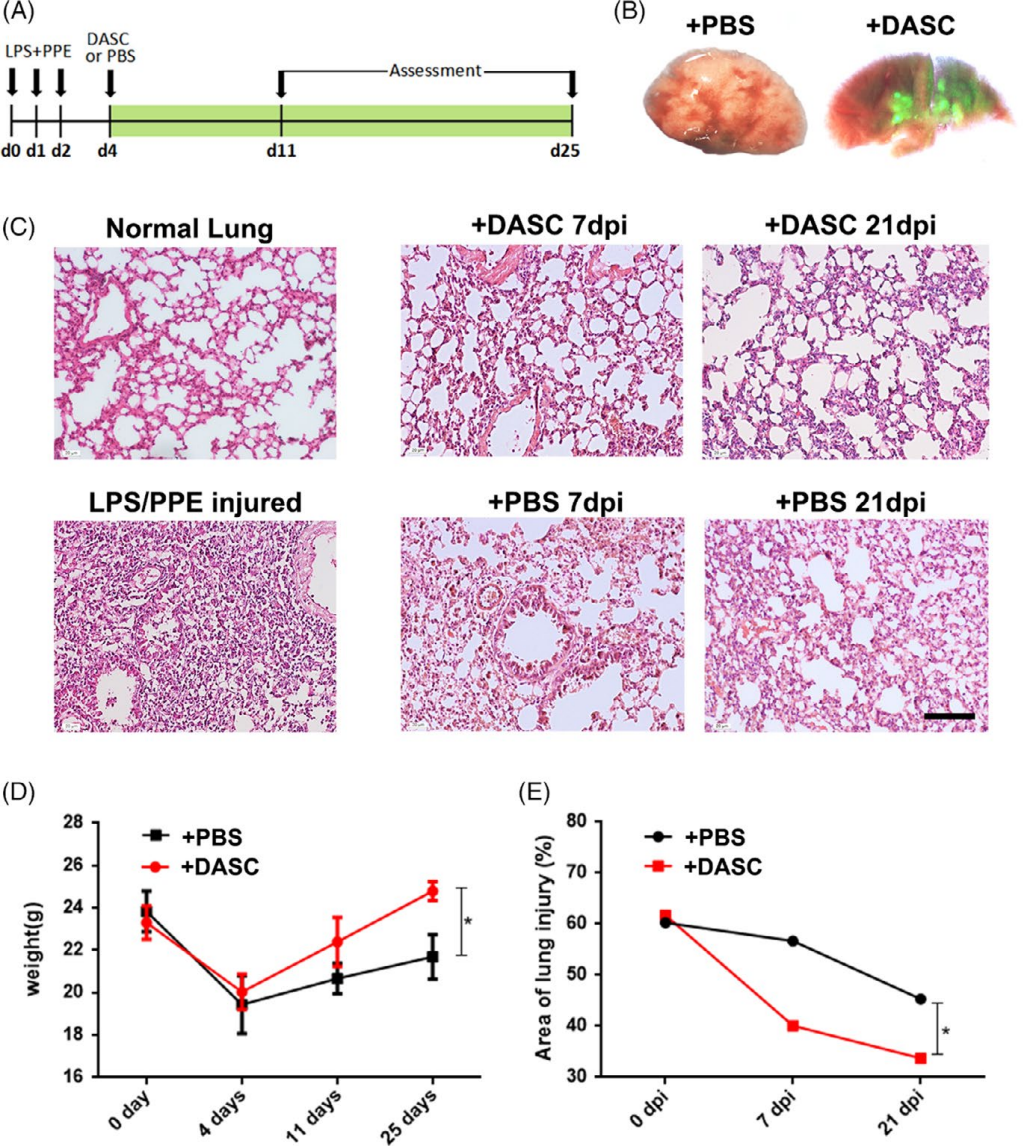
Intratracheal transplantation of human DASC to COPD mice. (A) Schematic diagram of the experiment design. (B) Bright‐field and direct fluorescence image of mouse left lung lobes following transplantation of 10^6^ GFP‐labelled human DASCs on Day 11. (C) Representative H&E staining of the COPD lung section after transplanted with cultured DASCs or PBS. The COPD mice were pre‐treated with PPE/LPS intratracheal administration to induce injury. Scale bar, 100 μm. (D) The weight changes of COPD mice after DASCs or PBS transplantation. N = 4. Error bars, S.E.M. *P < .05. (E) Quantitation of the injury area of the lung. *P < .05

To explore the potential therapeutic effect of DASCs in COPD lungs, human DASCs isolated from clinically diagnosed COPD patients were transplanted into the COPD mice lung (Figure 7A). The DASCs were labelled with GFP and cultured in a feeder‐free condition for one passage before transplanted into the PPE/LPS treated lung. Seven days after the cell transplantation, large scale of GFP+ cell incorporation was observed in both airway and parenchymal areas (Figure 7B). After administration of PPE/LPS, H&E staining showed that the degree of immune cell infiltration and alveolar collapse were alleviated post‐transplantation (Figure 7C). The reduced injury in the DASCs‐transplanted group was further confirmed by body weight and quantification of the damaged area in the lung (Figure 7D and 7E), indicating the pulmonary structure was restored by DASC transplantation.

**FIGURE 8:**
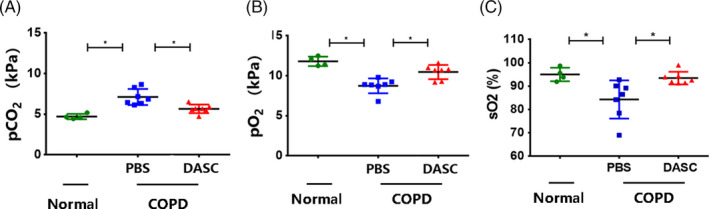
The pulmonary function of COPD mice can be restored by DASCs transplantation. (A‐C) pCO_2_, pO_2_, and sO_2_ levels of arterial blood after mice were challenged by PPE/LPS with or without DASCs transplantation. N = 4. Error bars, SEM *P < .05

Moreover, arterial blood gas levels were measured to assess the pulmonary function recovery after DASC transplantation. Consequently, compared with COPD lungs treated by PBS instillation, the mice treated with DASCs demonstrated higher O_2_ partial pressure (pO_2_) and O_2_ saturation (sO_2_), yet lower CO_2_ partial pressure (pCO_2_) in their Arterial blood (Figure 8A‐8C). Altogether, the existing data showed that the DASC engraftment could effectively restore pulmonary structure and improve the pulmonary function in COPD mice.

## 4 DISCUSSION

This study confirmed for the first time that transplantation of DASCs to COPD mice model could achieve lung regeneration and improve pulmonary function. Tissue‐specific stem cells have been identified as pluripotent cells with the capacity to renew and differentiate into other cell types. These cells are usually static under normal conditions and active after tissue injuries.^25^, ^26^, ^27^ Endogenous adult lung stem cells play an important role in the dynamic balance and injury repair of epithelial cells, which has attracted wide attention. Many human and animal lung studies have identified subsets of pulmonary epithelial cells with stem/progenitor cell potential, including bronchial basal cells and AT2 cells.^14^, ^28^, ^29^ In addition, advanced pedigree tracking techniques show that several types of pulmonary epithelial cells can proliferate and expand after injury to promote lung repair.^30^, ^31^, ^32^, ^33^, ^34^ A number of studies have shown that p63+/Krt5+ DASCs experienced rapid proliferation and migration to damaged alveolar areas in response to injury. These migrating cells gather and express typical alveolar‐related markers, suggesting that DASCs play an intermediary role in alveolar‐capillary network remodelling after lung injury.^16^, ^17^, ^18^

Different animal models have been developed to study the key mechanisms of COPD and to identify potential therapeutic targets. Several studies have reported that applying PPE into the lungs can lead to lung injury and inflammation, which leads to a chronic COPD process.^35^ Our results showed that PPE and LPS application caused significant lung injury within seven days, and the injury lasts to at least 21 days. In the current study, we observed that the incorporation of transplanted DASCs into the lungs of mice improved the lung injury and respiration of mice, showing an increase in oxygen saturation and a decrease in partial pressure of CO_2_.

Taken together, these findings suggest that DASCs might be involved in the regeneration and repair of lung injury. In addition, our results showed that five days after PPE/LPS infusion, DASCs improved the histopathological characteristics of lung tissue and prevented the development of pulmonary inflammation. Therefore, we discussed the protective mechanism of DASCs on COPD induced by PPE/LPS. It is consistent with this obvious protective effect of lung function. We cannot rule out the possibility that DASCs might play an indirect role in the progression of COPD by inducing other molecules. For example, DASCs have an immunomodulatory effect and may affect the infiltration of CD45+ inflammatory cells in the lungs. Therefore, DASCs may play a variety of roles (regeneration and/or immune regulation) in the process of COPD, and its specific mechanism remains to be further studied.

## CONFLICT OF INTEREST

All authors declare no conflicts of interests.

## AUTHOR CONTRIBUTIONS

Wei Zuo and Jieming Qu designed the study. Xiaofan Wang, Yu Zhao, Dandan Li, and Yueqing Zhou were involved in data collection and analysis. Yujia Wang performed the data analysis and prepared the manuscript. Yusang Xie, Yun Feng, and Min Zhou collected and provided the human bronchoscope samples.

## Supporting information

Supplementary Material

## Data Availability

The data that support the findings of this study are available from the corresponding author upon reasonable request. The scRNAseq raw data was uploaded to GEO datasets and the GEO number is “GSE168573”.
