# Supplementary material for: Intrapulmonary distal airway stem cell transplantation repairs lung injury in chronic obstructive pulmonary disease
Source: Cell Prolif. 2021 May 7;54(6):e13046. doi: 10.1111/cpr.13046 (PMC8168420; doi:10.1111/cpr.13046)
Supplement: Supplementary file 1 — Supplementary Material [file CPR-54-e13046-s001.docx]

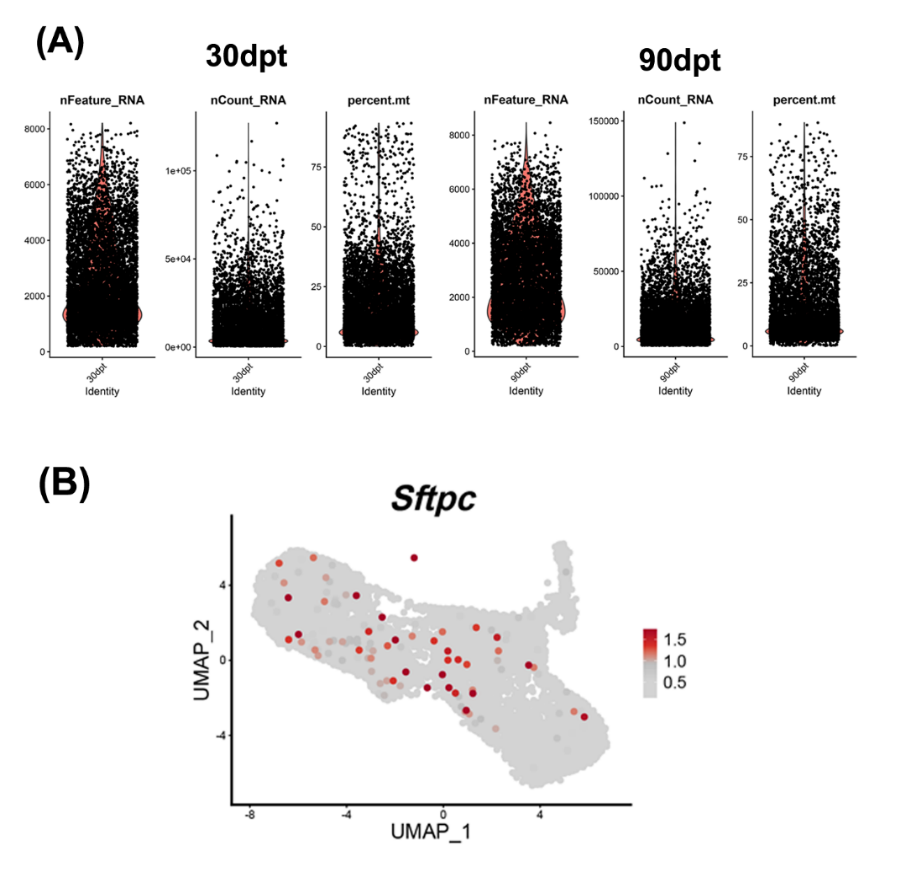


**Supplementary Figure 1. Quality control (QC) and expression of AT2 cell marker of the scRNA-seq data.**

(A) Scatterplots illustrating the number of genes, unique molecular identifiers (UMIs), and the percentage of mitochondrial genes in each cell of 30-dpt and 90-dpt sample.

(B) FeaturePlot highlighting the expression of AT2 cell marker SFTPC in the sorted cells.


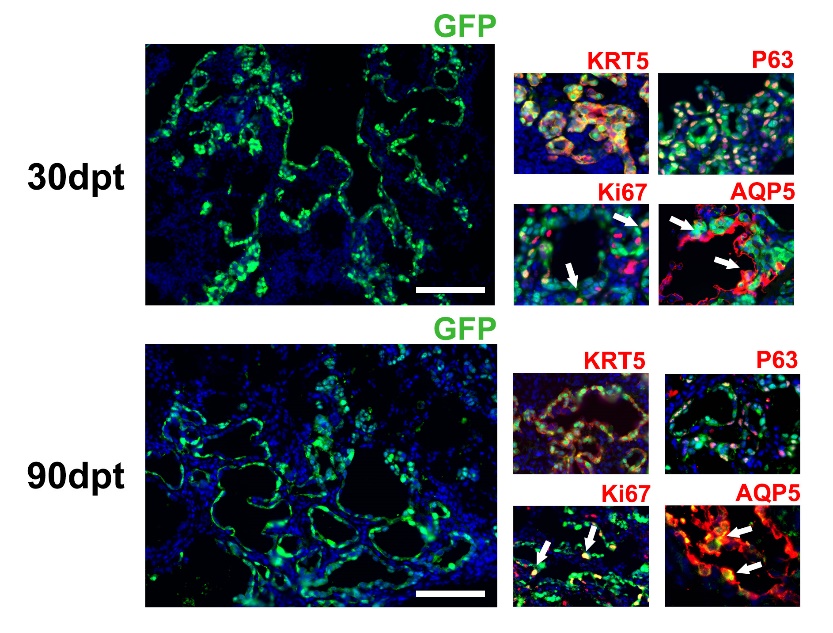


**Supplementary Figure 2. Histological characterization of the transplanted DASCs in vivo**

Immunostaining assay of KRT5/P63 (DASCs), Ki67 (proliferating DASCs), AQP5 (AT1 cells) and GFP (engrafted cells) in transplanted lungs at 30dpt and 90dpt sample. White arrow indicates co-expressing cells. Scale bar, 50 μm.

**Supplementary Table 1. Demographic and clinical characteristics of the COPD patients**

| Demographics |  |
| --- | --- |
| N subjects | 21 |
| Age (years) | 61.10±8.91 |
| Male sex | 21 (100%) |
| Body mass index (kg/m^2^) | 21.34±3.08 |
| COPD characteristics |  |
| GOLD 2001 status |  |
| II | 4 (19.0%) |
| III | 6 (28.6%) |
| IV | 11 (52.4%) |
| FEV_1_ (% predicted) | 32.81±13.31 |
| FVC (% predicted) | 58.30±16.57 |
| FEV_1_ /FVC | 54.58±9.46 |
| DLCO (% predicted) | 43.41±22.44^a^ |
| 6-minutes-walk (m) | 368.7±154.1^b^ |
| SGRQ score | 48.03±15.09^b^ |
| MEF25 (% predicted) | 15.17±4.74 |
| MEF50 (% predicted) | 10.14±5.14 |

Abbreviations: GOLD, Global Initiative for Obstructive Lung Disease spirometry criteria; FEV_1_, forced expiratory volume in 1 s; FVC, forced vital capacity; DLCO, diffusing capacity of the lung for carbon monoxide; SGRQ, St. George’s Respiratory Questionnaire; MEF, maximal expiratory flow-volume.

^a^Data from 16 patients were collected.

^b^Data from 18 patients were collected.
